# Hospital and Institutional News

**Published:** 1914-02-28

**Authors:** 


### HOSPITAL AND INSTITUTIONAL NEWS.

### SALVARSAN WARDS IN AUSTRALIA.

In view of the recent decision of the London Hospital to open salvarsan wards, it is depressing to learn from our Australian correspondent that the special wards for the treatment of venereal diseases—men at the Alfred Hospital and women at the Women's Hospital—have been closed owing to the Government finding the expenditure not justified by the results. Evening clinics for men had been proposed, and the matter was under consideration by the committee of the Melbourne Hospital, but the medical staff did not recommend it, Dr. William Moore remarking bluntly that if the men were well enough to work they could pay for private treatment; if not well enough to work, they could attend at the hospital in the day-time. The above decision is too summary to yield a moral for English institutions, which may therefore not be discouraged by this apparent failure in Melbourne. We must make our own experiments, and in the absence of detailed reasons conclude that the conditions attaching to admission to these wards were such as to destroy their value.

### INTERRUPTION AT AN ANNUAL MEETING.

A lively tone was given to the annual meeting of the Bristol Royal Infirmary last week by the speeches of certain Labour representatives who indicted the present system and contrasted it unfavourably with that prevailing at Leicester and Newcastle, and stated that the reduction of the working-men's subscription was a symptom of their dissatisfaction. Alderman F. Sheppard began the discussion by saying that the workpeople thought, as they were expected to subscribe, they ought to have a voice in the management, and that the promotion of a committee in Newcastle had led to an increase in the working-class subscriptions. Mr. Senington then made allegations—which, on the request of Sir George White, the president, he declined to specify—that things were not right, and said that the workpeople's representation alone could remedy them. Mr. Widdicombe followed to the same effect, saying there was a feeling abroad that the workers did not get fair play for the money subscribed. If they could not get representation they would withdraw their subscriptions. Representation would allow a fair opportunity for investigating complaints.

### "NO FIGHTING IN THE BOARD ROOM."

In reply, Sir George White said that Bristol compared favourably with other towns in hospital management, that Mr. Senington, judged by press reports, had initiated his movement with considerable acrimony, winding up with aggressive language. This spirit did not commend itself to him; they were not going to have fighting in the board room; and, according to the rules, any person who wished to displace the management should do so constitutionally by persuading one or two thousand governors to elect them in place of the present committee. After a further point on the voting had been raised in connection with the election of officers, Sir George White said that they were not going to allow that sort of interruption to take place at future meetings. Naturally exasperated at the tone of certain speakers, Sir George, in our opinion, hardly made full use of his opportunity, since his reply did not deal specifically with the main question: How does the representation of the working-class subscribers at the Bristol Royal Infirmary compare with that of other industrial centres? It was unfortunate, in our opinion, that he or Mr. Budgett was not moved to answer this question, and that Mr. Jackson's suggestion for a small committee to inquire into the point fell to the ground.

### A DEFENCE FROM NORTH EVINGTON INFIRMARY

Stirred by the criticism of the inadequacy of the nursing staff at this infirmary in our columns and other papers, the Leicester Guardians have taken steps to justify themselves. They do so by pointing out that their staff compares favourably with that of other infirmaries. They state that in Gateshead, Hull, and Norwich there is 1 nurse to 13 patients, in Blackburn and Sculcoates 1 to 11, in Tynemouth 1 to $10\frac{1}{2}$, in Bury, Newcastle, Southampton, and South Shields 1 to 10, in Ecclesall Bierlow 1 to $9\frac{1}{2}$, in Bradford, Leeds, Sheffield, and Sunderland 1 to 9, and in Birkenhead, Burnley, and Stockport 1 to 8, whilst at North Evington the proportion is 1 nurse to $8\frac{1}{2}$ patients. A bald comparison of this kind is fallacious, because the adequacy of a nursing staff depends not only upon its numerical strength, but also upon the arrangement of the wards and the nature of the cases admitted. Apart, however, from this fallacy, it is obvious that the figures do not necessarily prove that the North Evington Infirmary is properly staffed. They may, and probably do, merely show that, bad as things are there, they are still worse at most of the infirmaries enumerated. The facts remain that the resident medical staff, who are in the best position to judge, are of opinion that the number of nurses is inadequate, and that it was proved in evidence before the Coroner that one nurse had charge of thirty patients, fourteen under three years of age, distributed through three rooms. Proper supervision and nursing under such circumstances is impossible.

### SANATORIA BEDS FOR INSURED PATIENTS.

The death of an insured tuberculosis patient who has been in receipt of sick benefit, sad as it is, is at least an improvement on the list of insured patients who have succumbed before the benefit which they required was available. And in the inquest at Bethnal Green which followed on the case in question last week, a point was made of which not much has been heard hitherto—namely, that the patients themselves sometimes put difficulties in the way of going to a sanatorium. The mother of the deceased told the Coroner that she had a grievance against her panel doctor, Dr. Jenkins, for not sending the girl away to a sanatorium. Dr. Timothy Heas, the partner of Dr. Jenkins, said that she had been unwilling to accept his advice and go into a sanatorium until her condition had become too advanced for such treatment to be of any benefit to her. The jury thereupon returned a verdict of "Natural death," and exonerated all concerned from the imputation of injustice. The mother's attitude is only explicable on the grounds of grief at her loss, and the feeling, which often lends a sting to grief, that all has not been done. What, however, had the sickness benefit amounted to? First five shillings a week, and later on four shillings. This, small as it is, is better than nothing; but the question remains, Could Dr. Jenkins have guaranteed her a sanatorium bed had she been willing to go before it was too late? Domiciliary treatment that cannot fall back on sanatorium beds is predestined to failure.

### THE SCANDAL OF DOMICILIARY BENEFIT.

THE Insurance Committee in London under the National Insurance Act has not been a success. It clearly wants reconstructing, and the clearing out of a number of its members who do not appear to have any adequate knowledge of existing institutions or of the cruelties persistently perpetrated on the insured. One of the latest scandals arises out of the invention of what is called domiciliary benefit. Two instances have been brought to our notice —one, a young woman with tuberculosis, who was attending at one of the great Metropolitan hospitals, and whose visits suddenly terminated without cause given, though her case was progressing favourably under treatment. Some weeks elapsed, and then the patient presented herself again, with the explanation that she had been notified that the Committee had granted her domiciliary benefit, which she accepted as a special kind of treatment. She found, however, it really meant attendance on a panel doctor, who merely gave her "a bottle of cough mixture." In the second case a young man had been attending the out-patient department of a consumption hospital in London for over a year because the Insurance Committee paid no attention to his urgent requests to be sent to a sanatorium. At last he received notice that domiciliary benefit had been granted to him, which turned out to be an out-patient note on another consumption hospital for three weeks, when he was instructed that he must obtain a further out-patient ticket from somewhere.

### MOCKERY BY AN INSURANCE COMMITTEE.

THE examples we have given of domiciliary benefit—and they multiply increasingly—condemn some Insurance Committees as incompetent and cruel. The Committees too often mock insured persons suffering from tuberculosis, and the scandal grows graver every week from the fact that an increasing number of insured persons who might possibly be cured are allowed, under this scandalous system, calmly fostered by those responsible for the administration of the Insurance Acts, to grow steadily worse, until the patients become so ill as to render recovery hopeless in a multitude of cases. Is there not one member of Parliament who will make it his business to ask that particulars of every case of the kind may be sent to him by the victims or their friends? He should then undertake to publish the circumstances of every authenticated instance of mockery by an Insurance Committee, until the Government is compelled to do its duty and make National Insurance in regard to tuberculosis cases efficient, or to suspend this section of the Act altogether. Under the Insurance Acts patients suffering from tuberculosis are often given a cruel existence, aggravated in every possible way by the mockery and incompetence of those responsible for the administration of the Acts. These shocking cruelties are the direct fruit of reckless politicians who have light-heartedly set up the present machinery of torture and destruction without a thought for these unfortunate sufferers and victims.

### THE CAMERON PRIZE FOR PROFESSOR EHRLICH.

THE Senatus Academicus of Edinburgh University have awarded the Cameron Prize to Professor Paul Ehrlich, by way of recognition for the discovery of salvarsan, for his researches into the synthetic compounds of arsenic, and his labours on the subject of immunity. The Cameron Prize is granted for "highly important and valuable additions to practical therapeutics," and redounds in the present case not merely to the famous professor, but to the institution and city of Frankfort, where his experiments have been carried on—namely, the Royal Institution for Experimental Therapeutics, of which Ehrlich is, of course, the director.

### A CHAIRMAN AS HOSPITAL HISTORIAN.

MR. EGBERT LEWIS, chairman of the board of the Bath Royal United Hospital, has been compiling in newspaper form a brief historical review of his institution. His object is to put the case for the hospital before the public, so that by realising the development of its work the public may subscribe more liberally. It was not till 1747, he states, that the need was felt for a hospital for the relief of casual sickness or accident among the poor generally, and the then institution, called the "Pauper Charity," boasted that it was the only one in Bath for the relief of sick poor in the city and its neighbourhood. Medical assistance was given either at the dispensary or at the patients' homes; but its weakness was the absence of accommodation for serious accidents. A casualty hospital sprung up in 1788, thanks to Mr. James Norman, until in 1792 a larger building was necessary, and the charity was reorganised as the Bath City Infirmary and Dispensary. In 1822 proposals for amalgamating the two institutions were made, and two years later the first stone of the older part of the existing hospital was laid. In 1826, therefore, the Bath United Hospital was ready for the reception of patients. The Prince Albert Wing was added in 1864, and the title

"Royal" was granted by Queen Victoria. In 1887 the then president, the Rev. Edward Handley, secured the old Hetling Pump-room as an institute for the private nursing staff, and in 1891 the children's ward was added as an upper storey to the Prince Albert Wing. A new laundry and observation wards were later added, together with a large kitchen and chapel in 1897. We have always welcomed hospital histories as a work which every institution should undertake, and Mr. Lewis is to be congratulated on presenting to the people of Bath the story of the efforts of previous voluntary workers.

### SANATORIA AND CONVALESCENT HOMES.

Mr. Charles Harding, J.P., has been elected President of the Royal West of England Sanatorium, Weston-super-Mare, which has had an eventful year from the closing of the institution, which virtually took place during its redecoration and the installation of the electric light. To meet this expenditure £1,000 was raised through the generosity and assistance of Miss Mawe, the hon. lady superintendent, who subscribed herself, helped to raise the funds, and lent her residence during the year for the benefit of the sanatorium. The resignation of this official, since withdrawn, and the death of the late honorary secretary, Mr. William Dealtry, also interrupted the smooth flow of the work, but Mr. J. J. Ranson, who kindly took his place, has consented to continue, and makes a satisfactory start with the institution's redecoration having been carried out without any encroachment on the ordinary revenue. It is noteworthy, too, that the work of the sanatorium is undergoing some change, from the fact put forward at the annual meeting by Dr. William Fligg, the medical officer, that fewer pulmonary cases are being received in consequence of the number of new sanatoria now opened, while the number of surgical cases tends to increase. But then, this institution, we understand, has never limited itself entirely to the various forms of tuberculosis. Since it is desirable that the latter disease should be treated in special institutions the tendency here noticed is in the right direction. There should be no ground for confusion between sanatoria proper and general convalescent homes.

### THE CRAZE FOR SPOON-FED LEGISLATION.

In another column of the present issue we direct attention to the threatened interference with the free gifts from working men to voluntary hospitals through the action of a few members of Parliament, including Lord Henry Bentinck, Mr. Buxton, and others. This attempt was buried in a Bill which aimed at a quite unnecessary amendment of the Truck Acts. There can be no doubt that the Bill as it stands would reduce the income from the industrial classes which they at present give voluntarily and cheerfully to the voluntary hospitals by probably one-half. This Bill would wantonly interfere with the free action of the working classes in making voluntary subscriptions or payments to "any benefit society, sick club, hospital or any other society, club, or benefit or for medical attendance." In the large centres in Scotland and the North of England a substantial proportion of the ordinary income of the hospitals is contributed by the industrial classes, and for the convenience of collection it is mutually agreed between the masters and workmen that these gifts shall be deducted at the pay office of the various mines, workshops, and establishments each week. This system of contribution to voluntary hospitals is exceedingly well organised, and has come to be regarded properly as a regular source of their income. The difficulty of collecting small sums by weekly payments is universally recognised, and the small weekly deduction, voluntarily given, the workman does not miss, though in the whole it amounts to a substantial sum for the hospitals each year. The House of Commons has lost most of its old reputation as a legislative assembly in the days when it was a representative centre of public opinion. This wanton Bill, which we are happy to hear has been withdrawn for the moment, is a striking example of the causes which underlie this loss of the confidence which all classes formerly had in the common sense and intelligence of the average member of the House of Commons.

### A GREAT OPPORTUNITY.

The British Hospitals Association is taking up actively the Truck Acts Bill, and it will no doubt approach Parliament by petition. The Hospitals Association would indeed have been lacking in its duty if it had not taken immediate action with a view to expose the far-reaching effects for evil of this latest craze for spoon-fed legislation. We hope the constituents of Lord Henry Bentinck, Mr. Buxton, and the other members of Parliament who have backed this Bill will soon make it plain to the M.P.s in question that they will have either to amend their ways or may cease to continue members of the House of Commons.

### THE ANSELL REVERSION.

The Ansell fortune of a quarter of a million, which, as we noted in our issue of January 3 last, by the death of a boy of seventeen became available for the hospitals, is again brought into notice by the granting of letters of administration of the boy's effects which have been sworn at £301 by his mother, Mrs. Grace Adèle Ansell, as the only next-of-kin. The boy's death as a minor, which was the occasion of this large sum becoming available for charity, appears to have been due to an accident which he met with two years ago on the Cresta run. Coming, as the bequest did, hard on the announcement of the Wernher bequest of nearly half a million to King Edward's Hospital Fund for London, the hospitals were placed in the remarkable position of receiving not far short of a million from two testators. It would be impossible to find a more convincing instance of the grip which the voluntary system has upon the affections of our fellow-countrymen.

### A CASE FOR INQUIRY AT CAMBERWELL.

At an inquest on a man who died in Constance Road Workhouse, Camberwell, the Coroner made some caustic comments, whilst the jury added to their verdict of "Death from natural causes" that there seemed to have been considerable want of attention. The man was raving on the day of his admission, and Dr. J. T. Rankin, the medical officer at the institution, gave instructions that he was not to be left alone without a male attendant. Unfortunately the Board of Guardians have not provided attendants for cases of this nature, and the attendant not only had to look after the raving patient, but also five or six other patients. Dr. N. E. Gilchrist, assistant medical officer at the workhouse, who forwarded the particulars to the Coroner and made the *post-mortem*, stated that he was told by the ward attendant that the man was dead. He did not then visit him, nor did he see the body until he made the *post-mortem* at the mortuary. The Coroner said that that was a slipshod way of doing business, likely to lead to great extra work on the part of his officer. It was bad enough in the case of anyone dying in a private house, but there was much more reason for care in a public institution. The Coroner added that there was no evidence to show that the doctor's instructions had been carried out, and he was bound to say that when "an attendant had five or six patients in his charge, and also a patient who demanded his whole attention, it did not seem proper." It is the duty of the medical officer to insist on the Guardians providing proper attendance. Have Dr. Rankin's recommendations been disregarded, or has he been slow to insist upon them?

### A NOTABLE CHAIRMAN'S RESIGNATION.

Sir Charles Seely, father of the present Secretary of State for War, has resigned the chairmanship of the monthly board of Nottingham General Hospital. Sir Charles, who is in his eighty-first year, has held the chairmanship for seventeen years, and his connection with the institution began as far back as 1869, when he first became a subscriber. He has also the distinction of having occupied the office of president more often than anyone else, and was first elected to this post in 1873. He was re-elected twenty-two years later, and held the office annually from 1900 to 1904, and again in 1911. The hospital is indebted to him not merely for personal service but for liberal contributions, notable among which is his gift of the cost of reconstructing and equipping the old wing, and of the new out-patients' department, nurses' home, and laundry, which were rendered necessary by the successful erection of a Jubilee Wing in 1897, the year in which he first became chairman of the monthly board. The convalescent home at Newhaven, consisting now of two houses separately acquired, have been purchased and maintained by him for the hospital's benefit. Other Nottingham institutions and charitable agencies have experienced Sir Charles' generosity, and he was presented with the honorary freedom of the borough in 1895. Simultaneously, moreover, the senior surgeon, Mr. Owen Taylor, who has been a member of the honorary staff for twenty-five years, and for the past eight has acted as its chairman, is also resigning his post.

### AN EXPERIMENT IN MEDICAL RELIEF.

The Local Government Board have approved of a scheme submitted by the Bermondsey Board of Guardians to reorganise the administration of medical relief in the parish. It provides for a whole-time medical staff, under the control of the medical superintendent of the infirmary, Dr. Bell. The Board are of opinion that the scheme should be regarded as experimental, and that the staff of assistants to the medical superintendent should be limited, in the first instance, to a term of five years. Two assistants are to reside in the infirmary, and be engaged solely on institutional work, and two or three others are to act primarily as district medical officers, and to reside in the district assigned to them, but also to devote part of their time to work in the institutions. The Board is also prepared to sanction the appointment of a part-time consultant in private practice. They think it very desirable that a whole-time medical staff, working under these conditions, should keep in touch as far as possible with outside medical experience. The Guardians are of opinion that the medical staff should not be limited to four assistants, but that there should be five. They have decided to increase the salary of Dr. F. S. Cream, first assistant medical officer at the infirmary, from £200 to £230 a year, and that of Dr. D. G. Eccles, second assistant, from £120 to £150.

### THE COUNTY AUTHORITIES AND WORCESTER INFIRMARY.

A discussion on the subject of providing attendance on suicide cases was held by the committee of Worcester General Infirmary last week. The city authorities, it seems, decline to make provision, while the county police always send an attendant. The secretary said that he had seen the Watch Committee on the matter, but without obtaining any concession. As we remarked in this connection on September 6, not everyone will agree with Dr. F. J. Smith's opinion that police watching is a nuisance to the hospitals, and should be done away. In another connection the chairman reported that the County Council desired the infirmary to accept occasionally tuberculosis cases which were suitable for treatment in the hospital. The committee recommended that the cases be received on a payment of £4 4s. for the first month, and £1 1s. per week for any further time. In reply to Dr. Neville Crow, the secretary said that the County Council must communicate direct with the hon. medical staff.

### THE PENALTY OF A DESPAIRING POLICY.

Those who followed our criticisms of the financial policy of the infirmary authorities last year will hardly be surprised to learn from the chairman that the committee, on making up their estimates for the current year, feared that probably the income still would not cover the expenditure, notwithstanding the reduction in the number of beds. So far as they could see, the deficit would be about £1,000. If they were to carry on the institution on the limited scale now adopted, they would still have to sell out funded property to the amount of £2,000. The Finance Committee recommended the governors to give authority to sell out stock to that amount if it was needed. The motion was carried. The Chairman also said that the matron reported that she could not get probationers, and she thought it was because no salary was offered. He moved that a salary of £5 be offered for the first year. After some discussion, and regret that an outlay of about £50 per annum should be incurred, the motion was carried. Even the most unrestricted admirer of the present policy can hardly feel much satisfaction at its fruits. The closing of beds and the selling of stock are only hostages to fortune; and is it surprising that probationers are fighting shy of an institution which is, as it were, slowly ceasing to exist? A really energetic campaign for raising funds in the county would be the best cure for all these ills.

### THE TRAINED NURSES' ANNUITY FUND.

We have received a circular-letter of appeal, signed by the Countess of Hardwicke, Lady Barlow, and others, which appears to have been addressed to the authorities and matrons of hospitals and other institutions employing nurses throughout the country. It asks them to co-operate in organising a contribution of a penny a month from every member of the nursing profession to form a Benevolent Fund, and to represent "the nature of such a fund as this to their nursing staff." The employers of nurses are asked to retain one penny a month from the salary of each nurse who consents to make this contribution, or to appoint one of the sisters as collector on behalf of the Trained Nurses' Annuity Fund, and to co-operate in organising a sale during the year. It is not quite clear whether this circular has been addressed to the authorities of Metropolitan institutions only at present, but a wide extension of the scheme in future years is clearly indicated. Lady Hardwicke and those associated with her are evidently labouring under some serious misapprehensions.

### PROPOSALS IT IS WISE TO ABANDON.

They appear to be ignorant of the Junius S. Morgan Benevolent Fund in connection with the Royal National Pension Fund for Nurses, of which Queen Alexandra is president, to which a very large number of the nurses throughout the country contribute already one shilling a year. This Benevolent Fund has been in existence for nearly a quarter of a century, and it has done and is doing an immense amount of work for all types of nurses. Its income amounts to quite £1,500 per annum, and the sum expended has steadily increased year by year from the outset. There is, therefore, no necessity for a second Nurses' Benevolent Fund in this country, nor any justification for troubling hospital officials and nurses to duplicate the work as suggested in Lady Hardwicke's circular. We may further point out that it was clearly understood when the payment of the Nightingale annuities was entrusted to the Trained Nurses' Annuity Fund that this fund should remain a neutral body and not enter into competition with existing organisations. We hope to hear that the scheme embodied in Lady Hardwicke's circular, which we believe to be due to an ignorance of the facts, has been abandoned. Working nurses throughout the Empire owe first allegiance to the Royal National Pension Fund for Nurses, and are justifiably proud of the great Junius S. Morgan Benevolent Fund which has been organised and maintained largely from the outset with the spontaneous and continuous help of British matrons, sisters, and nurses.

### RETIREMENT OF A HOSPITAL SECRETARY.

The Monkwearmouth and Southwick Hospital has lost an old friend with the resignation of Mr. J. G. Jordan, who has decided to give up the post of honorary secretary. Mr. J. W. Proom, the chairman of the finance committee, remarked, at a meeting of the governors last week, that Mr. Jordan had "practically grown up with the place" and had an almost unique experience of the institution. Mr. Jordan was publicly thanked, and elected a vice-president for his services. The institution was enlarged to its present size of forty-three beds in 1896, and a movement is on foot to construct a new hospital, for which, we understand, funds are now being collected. Mr. Jordan leaves, therefore, at a time when new developments are in view, and the growth of income is on the whole encouraging. The administration comprises a clerk and collector, Mr. G. W. Thompson. Is it in view of future changes that it was decided not to appoint another honorary secretary?

### A SUDDEN INCREASE IN HOSPITAL EXPENSES.

An inquiry is to be held into the great increase of expenditure which has been incurred at the Coventry and Warwickshire Hospital for the three months of November, December, and January. While the income for this period has increased by £133, the expenditure has increased by £1,100—namely, from £1,670 to £2,763. The Chairman Mr. W. J. Wormell, said at the monthly meeting last week that this increase was abnormal, but that the analysis before him was incomplete. It showed, however, that the item headed "Domestic" had increased from £325 to £868, and as far as the analysis had gone at present, £300 had been spent on bedding and replacements, as compared with £50 last year. The coal bill, too, had gone up from £159 to £273. About one-third of the expenditure, therefore, remains to be accounted for; the increase in heating expenses had been expected owing to the extension of the hospital. The committee agreed to defer the matter on the understanding that a report would be issued. As the Coventry and Warwickshire Hospital is one of the provincial institutions which has adopted the Uniform System of accounts, the inquiry should be very easily conducted, and, indeed, it is a little surprising that all the facts could not be put upon the table at once. As to the linen, an inventory made every six months is a remedy almost too obvious to insist upon.

### DOVER COAL.

From a very amusing speech made the other day by Sir William Crundall on behalf of the Royal Victoria Hospital, Dover, it would seem that the local collieries are something of a joke in the neighbourhood. Sir William remarked that the Stonehall Colliery contributed £25 to the hospital, and added that he thought this a good example, and that the other collieries within a nine-mile radius of Dover might render material financial assistance. Beyond that, he added humorously, the collieries might as well make up their minds to find the hospital in coals for the whole year. If they did this between them it really would not make much difference to them, especially when they reached that seam which they were now so rapidly approaching, and which consisted of good household coal. There was much good-humoured laughter at this, and if the principle be accepted it might be extended. For Dover is famous for two very useful commodities: its coal and its soles. Could not the hospital be supplied with these also?

### NEW CHAIRMAN OF THE SATURDAY FUND.

Everyone will sympathise with, and at the same time regret, the decision of Sir Savile Crossley to retire from the chairmanship of the Hospital Saturday Fund, which was announced at the first meeting of the reconstituted Board of Delegates last Saturday. He has served for fourteen years, and during that time the Fund has maintained a steady advance, which, as he pointed out, received a check only with the introduction of the Insurance Act. The Hon. H. L. W. Lawson said that during Sir Savile's chairmanship the total collected had increased by 50 per cent., and the amount received by the Surgical Appliance Committee and the Distribution Committee had increased, he believed, ten times over. Sir Savile moved that Sir T. Vezey Strong should be appointed his successor, Mr. Harry Gower seconded, and the motion was carried. Sir Savile Crossley stated that the King had expressed his approval of the new appointment. The Fund has experienced its difficulties and controversies of late years, but we think that the attitude finally adopted at the latest of these, over the almoners question, when it was finally recognised that the hospitals could not be dictated to in the matter of recommendations, a good augury for the future, and for the harmonious working of the Saturday movement under the chairmanship of Sir Vezey Strong.

### A HOSPITAL SECRETARY'S SAVINGS.

The sort of person from whom one does not immediately expect a legacy to hospitals is a hospital secretary whose life has been spent in collecting them or donations from others. But an example of a rare occurrence is provided by the latest accounts of Tavistock Hospital, to which the late Mr. T. F. Pearse, who had been for several years honorary secretary, has bequeathed a legacy of £45. The idea is, as Mr. R. Morshead, chairman of the committee, has explained, to expend it on the improvement of a ward kitchen and a pantry on the upper floor of the building. Another sum received requiring special mention is the gift of £25 from one who sends it "in lieu of a legacy," and who has sent it so regularly as to have given the hospital a sum total of £300. It will be noted, moreover, that the late Mr. Pearse was honorary secretary. It is to be feared that the secretary proper can hardly amass much savings for charity.

### CLOSING OF HALIFAX EYE HOSPITAL.

The closing of the Halifax Eye, Ear, and Throat Hospital is now an accomplished fact, and, no doubt to the advantage of the patients and the institutional world of Halifax, the work for which it was responsible has been transferred to the Royal Infirmary. In our issue of August 9 last year we detailed the work which, begun by Dr. John Oakley in 1886, and carried on by him and his son until the quarters became recognised as quite unfitted for modern hospital treatment, has been accomplished, and foreshadowed the closing which took place on December 31. The final general meeting of the subscribers was held last week, presided over by Mr. W. S. Milligan, the president, who pointed out that the Insurance Act had relieved them of patients, and recommended that the balance in hand of £150 should be handed over to the Royal Infirmary, to which, as carrying on the work, he urged the Eye Hospital's former supporters to subscribe. After these proposals had been approved, Dr. Oakley remarked that there had not been a single death, and that not more than half-a-sovereign had been spent on alcohol during the twenty-eight years. A vote of thanks to the president and to Mr. F. S. Mitchell, the hon. secretary, was passed, and the latter had thus the rare experience of presiding at a final annual meeting.

### THIS WEEK'S DRUG MARKET.

Few change in values have taken place during the week, and business on the whole has continued quiet, although there has been a fairly good demand for several articles. A somewhat large business has been done in quinine, and an advance in price at a not very distant date would not come as a surprise. Quotations for essence of lemon, which had been steadily dwindling down for some short time, somewhat unexpectedly advanced; but the general opinion is that it is doubtful whether the value will be maintained. Menthol is attracting less interest, and the tendency of prices is downwards rather than upwards. The value of opium is now more firmly maintained, and morphine has a slightly dearer tendency. Prices for cod-liver oil are rather lower, both for new season's and last season's oil; there has been some improvement in the fishing conditions, and the catch has been better. Jalap is selling at fairly low prices. At the public sale of drugs recently held in Mincing Lane there was a fair demand, but fluctuations in prices were few. Gum benzoin had a higher tendency; honey was in fair demand; sarsaparilla was rather dearer

